# Cultivating change: Addressing shifts in knowledge and skills required for landscape-scale nature recovery

**DOI:** 10.1007/s13280-026-02404-z

**Published:** 2026-04-26

**Authors:** Joshua Davis

**Affiliations:** Countryside and Community Research Institute (CCRI), University of Gloucestershire, Francis Close Hall Campus, Swindon Rd, Cheltenham, GL50 4AZ UK

**Keywords:** Capacity building, Knowledge systems, Landscape-scale, Nature recovery, Skills development

## Abstract

Delivering landscape-scale nature recovery depends on the effective acquisition and development of skills across agricultural, environmental, and governance contexts. This study draws on 25 semi-structured interviews with farmers, land managers, and intermediary organisations (delivery partners, bridging organisations, extension/advisory services) across England to examine how skills are acquired and developed in practice. Informed by taskscapes literature and structured around a novel five-domain analytical framework—systems thinking, lifelong and life-wide learning, collaborative partnerships, agri-environmental entrepreneurship, and technical expertise—findings highlight the centrality of collaborative, entrepreneurial, and technical capacities. They also reveal core tensions and misalignments: between (1) policy ambition and institutional capacity; (2) fragmentation of learning pathways caught between standardisation and flexibility; and (3) entrepreneurial initiative within compliance-oriented governance structures. The research underscores the need for tailored skill frameworks spanning formal, informal, and non-formal learning pathways to support adaptive knowledge exchange and the practical delivery of landscape-scale nature recovery.

**Supplementary Information:**

The online version contains supplementary material available at https://doi.org/10.1007/s13280-026-02404-z.

## Introduction

*“There is a disconnect between what we are asking people to do, and the way in which we are asking people to do it” (I16).*

Ambitions for nature recovery are gaining pace across the UK and globally, marking a shift in agri-environmental governance from piecemeal conservation to transformative land-use change (Turnhout et al. 2021; Wojtynia et al. 2021). Yet delivery remains inconsistent, and questions of who holds the knowledge, skills, and capacity to enact change are often overlooked (Scoones et al. 2020; de Boon et al. 2022a). In England, this transition intersects with a policy landscape in flux. Post-Brexit withdrawal from the Common Agricultural Policy has triggered wholesale reform of ambitions and incentives (Dewally et al. 2025), including the Agriculture Act (2020), Environmental Land Management Schemes (ELMs), 25 Year Environment Plan and Environment Act targets, alongside broader net zero and ‘30 × 30’ commitments (HM Government 2018; Little et al. 2024). Recent evidence, however, suggests that current efforts remain insufficient in both scale and ecological impact (Natural England 2025).

Landscape-scale approaches—including (re)wilding initiatives, regenerative farmer clusters, and catchment-based models—are central to achieving these ambitions (Sayer et al. 2013; Hurley et al. 2022; Wynne-Jones et al. 2022; Baker et al. 2025). Operating across landholding and institutional boundaries, such initiatives seek to align ecological restoration with sustainable livelihoods: bringing together farmers, advisors, conservation organisations, and policymakers (Butler et al. 2015). Herein, ‘landscape-scale nature recovery’ refers to multi-actor ecological restoration efforts across diverse land uses (Menz et al. 2013; NatureScot 2025; Ockendon et al. 2025). While practices range, they share common characteristics: shared learning, adaptive management, collaborative governance, and long-term stewardship (Duff et al. 2009; Barkley et al. 2024). As a result, actors must negotiate trade-offs between current agricultural production and future environmental restoration, public and private interests, and competing knowledge systems (Loorbach et al. 2017; Petit and Landis 2023), reflecting broader polycentric governance challenges associated with coordinating action across complex socio-ecological systems (Ostrom 2010; Wyborn et al. 2019).

Whilst learning and innovation are critical to managing such complexity, the specific ecological, entrepreneurial, and socio-technical skills needed remain underexplored (Prager et al. 2017). Enduring weaknesses in the UK’s Agricultural Knowledge and Innovation System (AKIS)—including fragmented advisory networks, access inequities, and limited investment in peer-to-peer learning—compound the challenge (Ingram and Morris 2007; Sutherland et al. 2023a). Despite reform, emphasis continues to be placed on metrics and mechanisms, rather than the relational infrastructures (i.e. trust, legitimacy, shared purpose, and collaborative relationships) that enable change (Klerkx and Proctor 2013; Šūmane et al. 2018; Nettle et al. 2022). Landscape-scale initiatives demonstrate that knowledge exchange and capacity building are collective, iterative processes (Reed et al. 2016; Prager and Creaney 2017). Their effectiveness depends on peer learning and shared experimentation: features that remain underdeveloped in current advisory and institutional frameworks (Blackstock et al. 2021; Sutherland et al. 2023b). Therefore, new delivery models are needed that strengthen individual and institutional adaptive capacity (i.e. the organisations, norms, and governance arrangements shaping practice) to overcome persistent socio-political and structural barriers (de Boon et al. 2022b; Nye et al. 2023; Birchall and McDonald 2025).

This study presents findings from 25 semi-structured interviews with key actors, including farmers, land managers, and broader intermediaries (delivery partners, bridging organisations, and extension/advisory services) across England. It asks: (1) What transformative (high-level) and instrumental (functional) skills underpin landscape-scale nature recovery? and (2) how are these skills acquired and developed in practice? Building on Ingold’s (1993) concept of ‘taskscapes’—landscapes constituted through patterns of activity and practice—the paper advances debates on the social infrastructure of nature recovery. The work introduces ‘skillscapes’ to emphasise how landscapes are structured through the distribution, development, and enactment of knowledge and skills within perpetually in-process, socio-ecological contexts. Just as taskscapes are typically analysed via five factors, the work proposes a five-skill analytical framework to examine how change occurs: through what forms of expertise, by whom, and with what support mechanisms (Curry and Kirwan 2014; Löfqvist et al. 2022). In doing so, these findings address current knowledge exchange and capacity-building reforms, with implications for both England and international contexts (Wentworth et al. 2021; Beacham et al. 2023), and highlight practical implications for skills acquisition and development.

## Conceptual background and analytical framework

This study explores how changing knowledge and skill systems support England’s transition to landscape-scale nature recovery and land use. Bridging literature on agricultural knowledge exchange, environmental governance, and sustainability transitions, it explores the learning required to translate ecological ambition into practice (Pascual et al. 2022). This section provides contextual background, clarifies terms, and introduces the analytical framework.

### Beyond the farm gate: Collaborative landscape governance

Restoring biodiversity and ecosystem function depends on actors’ ability to work across ecological, institutional, and disciplinary boundaries. Thus, landscape-scale nature recovery is both an environmental and socio-technical challenge: requiring new knowledge, relationships, and skills. While past approaches often emphasised individual farm management or site-specific interventions, contemporary initiatives increasingly coordinate action at broader spatial and governance scales, spanning landholdings, catchments, and communities (Reed et al. 2014; Hurley et al. 2022). Research on agricultural innovation and environmental governance has long recognised the importance of social learning, reflexivity, and peer-to-peer knowledge exchange (Dooley 2020; Ensor and de Bruin 2022; Nettle et al. 2022). At the landscape scale, these activities take on greater significance in building the legitimacy, trust, and partnerships capable of aligning diverse land-use objectives and delivering coordinated ecological outcomes (Adams et al. 2016; Prager and Creaney 2017; Cole et al. 2023). However, persistent skills shortages limit the effectiveness of landscape-scale action (Nesshöver et al. 2017; Sutherland et al. 2023b). Whilst attention has focused on mechanisms of knowledge transfer, less is known about the skills that enable these processes, and how individuals and organisations acquire and apply expertise as governance shifts from field- to landscape-scale levels.

### Knowledge, skills, and institutional capacity

Knowledge and skills are distinct yet interdependent aspects of human capability. Knowledge encompasses both codified expertise and tacit, practice-based understanding, while skills reflect the ability to apply that knowledge in context through technical, interpersonal, and system-oriented competencies (Heiskanen et al. 2016; Lybaert et al. 2021; Sørensen et al. 2021). Both are dynamic and relational: produced through interaction with people, place, and practice, rather than held as static attributes (Frank et al. 2022). Herein, this research treats them as composite forms of agri-environmental capital—collective resources (knowledge, skills, networks) that allow individuals and organisations to plan, implement, and sustain nature recovery at scale. Such capital accumulates through formal, informal, and non-formal learning pathways, shaped by the structural contexts in which actors operate (Legatzke et al. 2025).

Understanding the skills required for landscape-scale delivery means situating them within wider knowledge and innovation systems. The Agricultural Knowledge and Innovation System (AKIS) has long provided a framework for understanding the actors, institutions, and processes involved in agricultural knowledge exchange (EU SCAR, 2012). Yet, traditional AKIS literature privileges productivity-oriented innovation and individual learning, focusing less on collective action and ecological complexity (Nettle et al. 2022; de Boon et al. 2022a). While effective in specific contexts, such models struggle to capture the emergent, negotiated, and place-responsive aspects of agri-environmental transitions (Ingram 2018; de Boon et al. 2022b). In practice, knowledge infrastructures remain fragmented and subject to short-term ‘projectisation’, with expertise commodified in private markets (Knierim and Ingram 2024). This contributes to skills gaps and governance challenges, particularly regarding knowledge co-production and embedding practice (Prager and McKee 2015; Nesshöver, 2016). Here, institutional capacity refers broadly to the ability of networks, partnerships, and organisations to support learning, experimentation, and adaptation under uncertainty. In the UK, fragmented advisory systems and declining public extension services have generated disparities in access to trusted, independent advice (Curry 1997; Sutherland et al. 2023a). These challenges are amplified at the landscape scale, where coordination requires shared norms and new governance structures (Chivers et al. 2025). As a result, multi-stakeholder initiatives often depend on intermediary actors such as facilitators, advisors, and cluster leads to bridge governance levels and integrate local and scientific knowledge (Ratner et al. 2022). The skills required for these bridging roles are distinct from traditional advisory competencies but remain undervalued in policy and professional development (Pascual et al. 2022).

### A five-domain skills framework for landscape-scale nature recovery

This study builds on a semi-structured literature review and interview data to develop a five-domain analytical framework of core skills required for landscape-scale nature recovery (Supplementary Information S1). Herein, the framework is applied as an analytical lens. Empirical findings further refine and extend its application for interpreting how actors engage with, and operationalise, landscape change. It identifies five interrelated domains of competence, outlined in Table 1. The domains, though analytically distinct, are interconnected. Systems thinking provides the conceptual foundation for understanding complex landscapes; lifelong learning enables adaptation and knowledge renewal; collaboration fosters shared purpose and legitimacy; entrepreneurship translates innovation into action; and technical expertise grounds change in practice. Together, they offer a holistic skillset that underpins the shift to integrated, landscape-scale stewardship. The framework also extends beyond AKIS by foregrounding the ecological and collective dimensions of learning and innovation. While applied empirically, the discussion also considers its theoretical implications: namely, how findings point to a reconceptualised Agri-Environmental Knowledge and Innovation System (A-EKIS) that encompasses ecological principles, adaptive governance, multi-actor exchange, and scalar integration.

**Table 1:** Summary of five-core skill domains identified as critical for landscape-scale nature recovery, outlining their defining features, illustrative applications, and key supporting literature

Core skills domain	Definition & emphasis	Example applications	Relevant citations
Systems thinking	Capacity to navigate complex, interdependent socio-ecological systems. Involves recognising connections and feedback loops, anticipating unintended consequences, and working with ecological and institutional dynamics	Designing interventions that account for system-wide trade-offs and cascading impacts	Heiskanen et al. (2016), Cerutti et al. (2017), Francis et al. (2017), Blackmore et al. (2018), Sørensen et al. (2021), Rastorgueva et al. (2023)
Lifelong & life-wide learning	Commitment to continuous, reflexive learning across formal, informal, and non-formal settings—developing skills through practice, experience, experimentation, and adaptation	Engaging with peer-to-peer networks, farmer-led trials, or participatory action research	Sayer et al. (2013), Francis et al. (2017), Charatsari and Lioutas (2019), Padel et al. (2020), Sørensen et al. (2021)
Collaborative partnerships & network building	Building trust, brokering relationships, enabling collaboration across boundaries—enabling collective action at landscape scale	Facilitating multi-stakeholder governance in catchment or landscape restoration	Heiskanen et al. (2016), Laforge et al. (2018), Charatsari and Lioutas (2019), Thomas et al. (2020), Sørensen et al. (2021), Rastorgueva et al. (2023)
Agri-environmental entrepreneurship	Capacity to innovate under uncertainty and trial new approaches—blending finance, policy, and practice in ways that align economic and environmental goals	Designing or piloting payment schemes for ecosystem services across productive agricultural settings	Becot et al. (2015), Dias et al. (2019), Pliakoura et al. (2020), Mayor et al. (2022), Rossi et al. (2023)
Applied expertise & knowledge integration	Place-based know-how that integrates sector-specific (ecological, agronomic, and land management) knowledge into practical decision-making	Tailoring farm management plans using ecological monitoring data	Curry and Kirwan (2014), Nguyen et al. (2014), Heiskanen et al. (2016), Sewell et al. (2017), Šūmane et al. (2018), Sørensen et al. (2021)

## Materials and methods

### Research design

This study adopts a qualitative design grounded in pragmatic constructivism, viewing knowledge and skills as socially constructed. Instead of seeking universal claims, the goal is a situated understanding of shifting skills dynamics and the changing architecture of agri-environmental support systems for landscape-scale nature recovery. Semi-structured interviews collected individual experiences and system-level reflections, allowing participants to interpret and prioritise issues on their terms. Key topics included knowledge flows, learning pathways, and adaptive capacity. As such, the research responds to calls for grounded, experiential accounts of how skills and knowledge systems evolve in support of agri-environmental transitions (Mills et al. 2017; Thomas et al. 2020). For broader ethical and reflexive considerations, consult the Supplementary Information (S1).

### Participant selection

Participants were purposively selected through stakeholder mapping of AKIS actors relevant to landscape-scale nature recovery. Existing research (Prager and Thomson 2014; Ingram and Maye 2020; Knierim and Ingram 2024) aided in updating institutional networks across public, private, and philanthropic domains. Participants were classified by primary organisational affiliation and functional role into three groups: (1) Primary Land Managers, (2) Intermediary & Advisory Actors, and (3) Policy & Delivery Partners (see Table 2). However, in practice, many held multiple roles—including technical advisory, facilitation, and landscape-level coordination—reflecting the hybrid nature of intermediary functions within emerging agri-environmental governance systems. Subgroup identifiers show role-specific functions and hybrid/boundary-spanning positions, while anonymised in-text referral codes (I1-I25) are used for attribution.

**Table 2:** Participant typology and anonymised codes. Individuals are grouped by primary organisational affiliation or functional role, with subgroup identifiers included to capture hybrid/boundary-spanning roles. Anonymised codes are provided throughout for reference, and identifiers for attribution of illustrative quotes

High-level group	Subgroup identifier	Participant ID	In-text referral code
**Primary Land Managers** (i.e. farmers, estate managers, foresters engaged in nature-based initiatives)	Senior farm manager (livestock)	N8xDfk	I1
	Land manager—agri-tech project developer	ZFWgi9	I2
	Forester	QDpTs6	I3
	Rewilder	XV1qPx	I4
	Farmer (mixed)—rewilder	Rp0RGx	I5
**Intermediary & Advisory Actors** (i.e. environmental consultants, NGO officers, farmer cluster facilitators)	Farm advisor—environmental consultant	F4JjuO	I6
	Farm advisor—NGO officer	sF0SpU	I7
	Facilitator—environmental consultant	Sz8Fu1	I8
	Habitat restoration—delivery lead	R1JI7F	I9
	Environmental consultant—independent	tad6Gp	I10
	Training & development manager—environment	F5qWGe	I11
	NGO—land management advisory lead	Bvp4qN	I12
	NGO—strategy & stewardship lead	9Xr0sM	I13
	NGO—nature recovery lead	b6xIGj	I14
	NGO—nature recovery technical lead	IGoik8	I15
	Charity general manager—woodland creation	sQ7Ovg	I16
	Non-profit director—skills	6x3SyZ	I17
	Land-use & skills consultant—freelance	UJyE9N	I18
	Academic	x4UrIG	I19
	Academic—farmer (livestock)	Zk3cqM	I20
	Academic—farmer (mixed)	fdOwl4	I21
	Campaigns coordinator—horticulture	QeQ2Jg	I22
	Charitable trustee—landscape-scale conservation advisor	LvK7SQ	I23
**Policy & Delivery Partners** (i.e. government agencies/departments, catchment partnerships, local authorities)	Professional body—policy lead (agriculture/horticulture)	szYWd5	I24
	Senior advisor—non-profit strategy lead (nature recovery)	L7PcEX	I25

Selection criteria aimed to capture diversity in the scale of operation, geographical context, land-use type, and knowledge-sharing roles. Participants were included if they (a) directly managed land, (b) advised or coordinated nature recovery initiatives, and/or (c) influenced related policy or delivery systems. Using purposive and snowball sampling via professional networks, targeted outreach, and subsequent referrals, the final sample comprised 25 participants from across England and Wales. This breadth was not intended for statistical representativeness but to capture diverse perspectives across governance levels, providing a foundation for examining skills development, learning systems, and institutional change.

### Data collection and analysis

Interviews were conducted online between July and November 2024, lasting ~ 45–90 min. All were audio-recorded, transcribed, and anonymised. Materials (i.e. semi-structured interview schedule, participant information sheet) were shared in advance to support participant reflection and reduce response anxiety (Haukås and Tishakov 2024). The interview guides (Supplementary Information—Appendices S2 and S3) covered participants’ professional backgrounds, experiences of landscape-scale delivery, perceptions of learning and capacity building, and evolving skill requirements across both knowledge users and providers. Analysis followed Braun and Clarke’s (2006) six-step framework. An abductive approach was employed, using the five-domain framework as an initial coding structure, whilst allowing inductive sub-codes to arise. Coding was undertaken in NVivo (v.14), with iterative refinement across analytic cycles. Patterns were assessed at both the semantic (explicit meaning) and latent (underlying assumptions) levels, allowing the framework to be empirically validated and conceptually elaborated. Attention was also directed towards the directionality of knowledge flows: who learns from whom, and how these dynamics shift depending on context and support.

## Findings

**Fig. 1:**
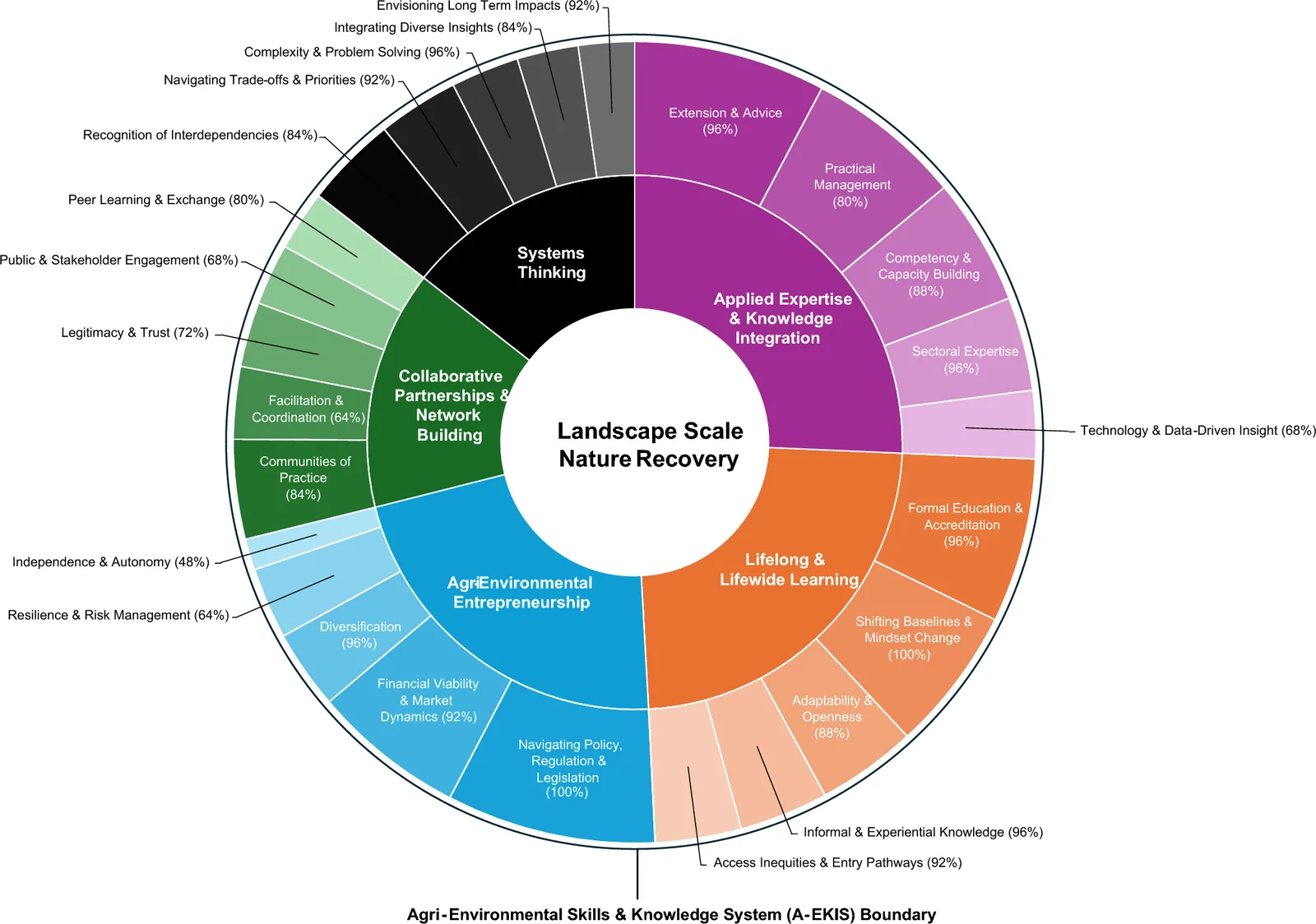
Five-Skill Framework. The inner ring illustrates the five interdependent skills domains: Applied Expertise & Knowledge Integration (Purple), Lifelong & Life-wide Learning (Orange), Agri-Environmental Entrepreneurship (Blue), and Collaborative Partnerships & Network Building (Green), and Systems Thinking (Black). The middle ring presents associated sub-skills—i.e. thematic codes derived from qualitative analysis of semi-structured interviews, while the outer ring visualises coded references from the interview dataset. Collectively, the analytical framework is situated within the broader Agri-Environmental Knowledge and Innovation System (A-EKIS), representing the institutional and knowledge infrastructures that enable learning and innovation for landscape-scale ecological transitions. *Note*: Slice size denotes the relative proportion of coded references associated with each sub-skill across the data corpus. Percentages indicate participant coverage—i.e. the proportion of interviewees who referenced the sub-skill (*n* = 25). This dual representation distinguishes between depth of discussion (reference intensity) and breadth of engagement (participant coverage).

The interviews reveal a dynamic and evolving skillscape. While participant responses aligned with the five pre-identified thematic domains (Table 1), inductive analysis identified further sub-skills that illustrate how these domains manifest in practice (Fig. 1). The following sections detail these findings with illustrative quotes and interpretive commentary: linking insights to broader debates on skills development, agri-environmental governance, and nature recovery.

### Systems thinking

Systems thinking—as a mindset and analytical tool—was seen as an essential skill for landscape-scale change: integrating farming, conservation, restoration, and rural development. Three central issues dominate: recognising interdependencies, negotiating trade-offs, and envisioning long-term impact.

**Recognising and acting on the interdependencies** between land, biodiversity, and livelihoods requires a shift from viewing farms or habitats as discrete units to understanding them as nested within dynamic, interlinked systems. This aligns with policy trends emphasizing landscape-scale governance, where ecological function, resource flows, and social relationships transcend farm and administrative boundaries. As one adviser noted, *“ecosystems are complicated… species don’t exist in a vacuum, but all of them have dependencies… you can’t squeeze nature into a five-metre strip*” (I6). Another echoed: “*There’s no hard edges in nature… it has to be joined-up, landscape thinking”* (I3).

Institutional fragmentation and siloed thinking limit integrated approaches. Agricultural, environmental, and rural development policies often operate through separate funding streams and metrics, limiting alignment of biodiversity, climate, and food security goals. Interventions in one area often cascade across others: at times productively, at times problematically: *“If you’re farming well for water and you’re farming well for carbon, you can’t help but be farming well for biodiversity”* (I5), whilst others warned trade-offs remain (I11). The challenge is to operationalise this without losing nuance: requiring systemic knowledge and skills to enact change. Participants called for multi-functionality in approach, aiming for balanced, joined-up solutions: *“You can do both… and it’ll work together in balance. But that doesn’t seem an option”* (I1).

Recurrent themes were **balancing trade-offs and navigating complexity**, often framed through dichotomies such as land sharing versus land sparing, or food versus nature. These simple oppositions were critiqued, with many noting the need for middle ground and context-specific solutions (I1). Yet, longstanding battlegrounds remain: *“From the word go, farmers were seen as the problem… that’s still an issue”* (I3). Equally reticent were issues of a *“them and us culture—it’s food or the environment… We need to get away from that… it needs to be locally contextualised”* (I15). In this context, systems thinking was not eliminating complexity but equipping actors with the adaptive skills to work within it. Participants advocated spatially targeted, context-sensitive approaches, acknowledging solutions must fit local realities, and that *“what works on one farm might not elsewhere”* (I19).

Systems thinking also involves **visioning for long-term impact**. Participants criticised short funding cycles and stressed the need for ongoing advice and realistic policy goals: *“People build relationships, feel we’re getting somewhere, then move on… Nature needs longer-term thinking”* (I4). Advisory support and foresight were seen as crucial: *“If you’re genuine about 30* × *30… you have to factor in land advice”* (I12). However, policy ambition remains poorly aligned with ecological and lived realities: *“2030 is a joke. We’re never going to reach that, not really”* (I7); and *“these are human stories… not just high-flung ideas about nature’s recovery. We need to respect that and support them as they go through this transition”* (I12). Ultimately, these perspectives position systems thinking as a relational competency that demands analytical insight and social sensitivity. It requires knowledge of interdependencies and sustained place-based processes that reconcile ecological ambition with the realities of nature recovery at scale.

### Lifelong and life-wide learning

“*We all need to keep on learning as we go: because there’s new knowledge, new circumstances, new contexts*” (I20).

As practices evolve, continual learning is essential. Participants described refining skills over time: drawing on *lifelong* professional development and *life-wide* experiences (i.e. formal education, peer exchange, and hands-on practice). Three themes dominated: unequal access to learning, shifting mindsets from farm-level to landscape action, and balancing diverse learning modes.

**Access inequities and entry pathways** shape opportunities to engage with nature recovery at scale: often influenced by geography, institutional ties, and social capital. Many felt excluded due to a lack of awareness or belonging: *“If you’re a farmer, or land user interested in engaging with nature recovery initiatives—where did you start? Who do you go to? What do you look at?”* (I19). This perpetuates unequal participation and creates a two-speed system where those with established networks expand their knowledge, while others risk marginalisation. Generational divides compounded this gap, with younger farmers more likely to engage with digital schemes and online offerings (I1). Here, the digitalisation of agri-environmental policy was seen to reinforce exclusion rather than enable participation. Scaling recovery, therefore, raises broader questions of capacity and justice: effective capacity building requires broadening points of entry and legitimising multiple knowledge systems.

Beyond access, **adaptability and openness** are crucial, as landscape change often reflects deeper cognitive and ideological shifts: “*The major barrier is education and changing people’s mindset…it’s more than just the training, it’s perception”* (I9). Transition is rarely immediate, often involving gradual reassessment and resistance: *“It’s a truism to say I do things on the farm the way my father did them, and his grandfather. Even the most reluctant farmers are now trying new things”* (I23). For some, change brought social friction and stigma: *“Our neighbours would shout over the fence, you’re not fucking country… look at the state of your land”* (I4), and *“everyone thinks you’re a bit mad, and no one really wants to talk to you about it”* (I1). These accounts underscore the importance of relational and emotive capacity—requiring tolerance for contested ideas and resilience.

Effective capacity building requires **diverse and interconnected learning pathways**: *“We talk about formal, informal and non-formal training… it’s not a curriculum, but a toolkit”* (I24). Informal and experiential approaches—working alongside peers or mentors—were seen as especially powerful: “*People learn through working alongside somebody who’s already done it… you overlay that with vocational qualifications; you’ve got the perfect model*” (I17). In contrast, formal education was criticised for being too narrow, with limited ecological focus: “*Not everybody wants to go to university… how do you make that pathway simpler? That’s a big challenge*” (I13). Yet, participants voiced concern over unaccredited training diluting professional standards: *“(we) find people are… setting up as independent trainers, but with no accreditation. Who’s ensuring you are delivering to appropriate standards, and what’s the qualification?*” (I6). Participants called hybrid models combining core curricula and national qualifications with non-accredited opportunities to ensure *“different learning pathways, towards a common goal”* (I3): supported by regional hubs for knowledge exchange and place-responsive skills training.

### Collaborative partnerships and network building

“*Landscapes aren’t competitive. They’re collaborative*” (I16).

Landscape-scale nature recovery depends on building trust, aligning interests, and facilitating joint decision-making, especially when institutional authority is diffuse, and co-benefits must be agreed upon. Two themes stood out: the importance of brokering legitimacy through facilitation, and the value of peer networks and communities of practice.

**Legitimacy, trust, and facilitation** were described as indispensable. Advisory roles were valued not solely because of technical expertise, but their ability to foster change: *“Collaboration itself is a skill… having the patience and know-how to work with difficult people”* (I22). This emphasis highlighted the importance of interpersonal and relational skills: managing power imbalances amongst actors. Here, top-down approaches undermine trust; engagement and local agency build it. Strong relationships form when actors set their own agendas and feel empowered: *“The best work we did was when we were told, what are your priorities?… It’s about being engaged rather than incentivised”* (I8), and *“everybody says peer-to-peer is the best: it has to be somebody they know and trust”* (I8).

Contributors emphasised **peer networks and communities of practice** in sustaining collaborative knowledge and learning. Farmers were seen to influence one another more than external advisers: reducing perceived risks and building confidence with new practices, effectively “*putting tools in the hands of the farmers to participate more fully in their own development”* (I6). However, sustaining these exchanges requires support. As one adviser reflected, “*the motivation to learn from others, and from the past: thinking fresh by reflecting… farmers train farmers, that is something they do*” (I19). Peer-to-peer processes, while organic, needed recognition and resources to scale effectively. Beyond informal exchange, participants highlighted the transformative potential of structured communities of practice. Responses for landscape-scale nature recovery—cluster groups, facilitation funds, and landscape recovery—were seen as varied avenues to enable farmers, advisers, and conservation professionals to share learning, access collective funding streams, and respond to policy incentives: *“The new kid on the block is the clusters… they’re dramatically changing the way that conservation messages are delivered”* (I14). Still, barriers remain: *“It’s difficult to get farmers to collaborate. Really difficult”* (I7). While some spoke positively of shared ventures, many noted unsuccessful endeavours and examples of formal cooperation remain rare. Incentives and facilitation support are vital to overcome obstacles and enable cross-sector skills gain: *“If you’re going to work at landscape-scale… there is no way you’re going to be able to make progress unless you cooperate”* (I6).

Collaboration, equally, extends beyond farmers and land managers. Several emphasised the importance of **public and stakeholder engagement** in monitoring and decision-making: *“We have to work in partnership… working with communities in terms of education and engagement… to identify the right interventions in the right places”* (I13). Collectively, collaboration is recognised as a core skill set, and democratic imperative: not an optional add-on, or a series of nice-to-haves. Trusted facilitation is necessary to sustain collective action, peer networks, and communities of practice, while broader community engagement embeds legitimacy across scales. In practice, collaboration requires balancing interpersonal skills with institutional support, ensuring the infrastructures that enable cooperation are prioritised alongside ecological goals.

### Agri-environmental entrepreneurship

Transitioning to nature-positive practices requires balancing ecological ambition with entrepreneurial risk: of which, financial viability is an essential prerequisite: “*You can’t go green if you’re in the red… We need to be looking at things in a very holistic sense*” (I5). Agri-environmental entrepreneurship extends beyond new practices, to encompass skills concerning decision-making autonomy, diversification of income streams, and the ability to navigate policy and markets.

**Independence and autonomy** were recurring issues: reflecting a common desire for control over land management decisions and avoiding reliance on prescriptive subsidies or AES: “*I want nothing to do with subsidies… which is why we’ve done rewilding, because we weren’t held to any schemes which told us what to do*” (I4). Others emphasised ownership of change: “*Ultimately, the destination belongs to the farmers; and, ideally, the funding that gets us there too”* (I8). Yet, autonomy was double-edged. While it allowed flexibility, it also risked disengagement from collaborative initiatives and longer-term funding opportunities. Advisers noted a lack of clear business plans, poor understanding of agreements, and variable uptake: *“These guys I work with—these bigger arable farmers—they know where they’re at. They’ve seen the value of stewardship and will do it”* (I7). This underscores the tensions between entrepreneurial independence and complex contractual landscapes, and suggest efforts are required to facilitate commercial skills development.

**Diversification** (i.e. eco-tourism, carbon and biodiversity credits, alternative business models) was widely seen as beneficial, and part of a broader cultural shift: “*There is a huge opportunity to diversify people’s income streams… and hopefully understand nature better at the end of it as well”* (I15), and “*farmers are entrepreneurial and will react and flex to change—if they don’t think they’re being forced”* (I25). However, diversification was not universally viable: farmers are seen as adaptable, but success depends on innovation, training, and support, and may not be viable in all geographies (I12). Thus, business skills—budgeting, project management, risk modelling—are essential tools for managing transition. An adviser explained: *“It’s the management skills; the business skills. It’s being able to look at—not necessarily the shiniest thing—but what’s most effective for your business”* (I24).

Across all interviews, **navigating regulation and novel markets** were highlighted as key factors shaping entrepreneurial potential. Frustration was expressed at a lack of knowledge, and *“overall element of uncertainty”* (I11) created by delayed or unclear government schemes: “*It’s over eight years since the decision to leave the EU… we don’t have any functioning ELM schemes”* (I20). Farmers described having to *“gamble”* on incomplete information when making business decisions (I11) and noted the disconnect between short political cycles and long-term ecological commitments (I2, I11).

“*The biggest skills or understanding block for the delivery of nature recovery is landowners knowing how to access funding… Outside of agricultural support, I don’t think it’s a reasonable expectation to do that. It’s so messy, so inconsistent, and there are so many players*” (I14).

Future markets offer promise, with blended finance and private investment opportunities; however, actors warned of risks, of uncertainty framed as “*a jungle*” or “*Wild West*” (I22), and of misaligned incentives: “*You’ve got nutrient neutrality, BNG… all these different policies aren’t aligned, and you end up with perverse outcomes*” (I15). Furthermore, stacking payments and aligning funding streams were also cited as major constraints (I13). Despite frustrations, initiatives like DEFRA’s Test and Trials, facilitation funds, and farmer clusters were praised for co-design and collective problem-solving (I22).

Concerns regarding **risk, resilience, and strategic adaptation** run throughout. Entrepreneurship was seen to involve not just seizing opportunities but developing strategies to cope with uncertainty: “*How do you future proof? … resilience and adaptation are going to be fundamental”* (I16). This requires balancing immediate economic needs with longer-term environmental goals, amid volatile regulatory and market conditions. As one adviser concluded: “*Most farmers are stretched to the limit… to throw a completely different load of legislation and a new market at them—when they have no experience of it—it’s scary”* (I3).

### Applied expertise and knowledge integration

Landscape-scale nature recovery requires technical knowledge alongside ecological understanding, spanning habitat management and species reintroduction, to soil restoration and monitoring techniques. This ‘applied expertise’ may be grounded in scientific training, lived experience, or traditional ecological knowledge and must be embedded within broader advisory and support systems to be scalable. Three cross-cutting themes were of relevance: translating practical management into ecological contexts, competency and capacity building, and the evolving role of advice and extension.

At its most immediate, participants emphasised **practical management** skills—on-the-ground decision-making, sectoral norms, and embedded expertise gained through years in a particular domain—as essential for landscape interventions. One adviser noted, *“we see most of those changes through the introduction of nature-based solutions… Planting trees or hedges in the right places, building wetlands, re-wiggling rivers, putting in buffer strips, working with farmers to improve their soil health”* (I13). While farmers and land managers often possess these skills, they may lack frameworks to apply them to ecological outcomes (I14). This challenge was particularly felt around monitoring and compliance. Increasingly, farmers are expected to benchmark and demonstrate biodiversity outcomes, often through unfamiliar surveys or audits (I11). This highlights a systemic tension—practical know-how exists, but its application to ecological restoration at scale requires new forms of integration and interpretation—seen as shifting but continuous with the ethos of ‘good farming’, and of conservation practice—i.e. “*doing the right thing in the right place”* (I14), and of “*borrowing from the past to create a new future*” (I17). As one interviewee stated*: “We need to be teaching people how to observe and understand their landscape in a way that works with the practices they do anyway”* (I16). Participants acknowledged the growing role of technology in this space (drones, no-fence collars, mobile apps, AI) but stressed that while digital tools could “*speed things along a bit*,” they did not replace the need for ecological literacy (I2).

More broadly, participants consistently emphasised the scale of **competencies and capacity-building** at both individual and institutional levels. Skills are provisional. In this way, expertise was viewed as iterative and adaptive: framed as a “*never-ending list”* (I3), and “*constantly moving skill set”* (I17). While nature recovery experts are therefore needed across domains, many acknowledged such roles are rare due to the breadth of required knowledge (I15). Again, this points to a broader structural dilemma: while the knowledge base for landscape-scale nature recovery is expanding rapidly, mechanisms for cultivating and coordinating it remain fragmented (I18). Capacity was also framed culturally: re-thinking what counts as expertise and resonating with broader debates on adaptive governance. This suggests capacity building must move beyond static training modules towards systems of continual re-learning that evolve with emerging challenges rather than plug immediate gaps.

**Advice provision and extension** emerged as essential yet underdeveloped components of the knowledge system: *“It’s not simply how to do nature recovery, but also how to advise on it”* (I11). Farmers are expected to act as better ecologists, but many lack access to trusted advisors to bridge technical expertise and on-farm realities. Here, the skills required extend beyond technical competence to relational brokerage. Effective advisors need to integrate ecological knowledge with sensitivity to farmers’ values: *“The first question you should ask is, what’s important to you as a farmer? … Those interpersonal skills are vital”* (I12). However, recruitment challenges, funding instability, and duplication of effort continue to undermine advisory capacity: *“Who offers advice where?… If you’re a farmer, and you want advice, where do you go? There’s a huge duplication of effort”* (I13). Promising initiatives, such as clusters and facilitation funds, illustrate how advisory roles could be embedded within trusted networks. For many, the core issue is not a lack of knowledge, but who holds it, and how it is brokered—highlighting the need for stronger connective infrastructure to address the apparent “missing middle” of advice provision (I10).

## Discussion

This study set out to explore the knowledge and skills required for landscape-scale nature recovery, and the mechanisms—formal, informal, and non-formal—through which expertise is acquired and developed in practice. Yet, what emerges is not simply an access or capacity gap. Whilst findings align with the current understanding of knowledge and skills (as composite forms of agri-environmental capital), they point to deeper structural constraints affecting how knowledge, trust, and resources circulate within and between individuals, institutions, and communities. In particular, the results highlight a series of systemic misalignments within current agri-environmental governance. Rather than discrete challenges, these tensions between policy ambition and institutional capacity, standardisation and learning flexibility, and entrepreneurial aspiration and risk-averse governance interlock and compound to influence how knowledge circulates, how learning is valued, and how innovation is incentivised. In short, they highlight where the system isn’t yet enabling the transition it hopes to support. The following sections unpack these dynamics and consider their implications for policy, practice, and theory.

### Working at the interface of policy, practice, and capacity

Notably, there exists a disconnect between the ambition of policy frameworks and the capacity of institutions tasked with their implementation. Respondents consistently described the pace and scale of initiatives—including Environmental Land Management schemes (ELMs), Local Nature Recovery Strategies (LNRSs), and 30 × 30 targets—as outstripping available skills-oriented, advisory, and institutional support. Frequent reforms and shifting priorities were seen to generate uncertainty and stasis, limiting the ability of local actors to plan, invest in new capabilities, or develop longer-term learning pathways. These findings reinforce wider critiques of environmental governance, which identify persistent mismatches in scale, institutional capacity, and administrative coordination (Waylen et al. 2015; Reed et al. 2022; Borniotto et al. 2025). However, the present study adds a further dimension by demonstrating how such governance gaps directly affect the development and distribution of key competencies required for landscape-scale action. In this context, systems thinking and integrative expertise are essential, yet the relational, technical, and adaptive capacities remain unevenly distributed across the agri-environmental landscape. These deficits are not incidental: in AKIS terms, they reflect a governance architecture that is insufficiently configured to foster knowledge exchange, reflective learning, and support the learning processes through which such capabilities emerge. Earlier work has highlighted the importance of such in supporting agricultural innovation (Klerkx and Leeuwis 2009; de Boon et al. 2022a). The present findings extend this insight by illustrating how gaps in these connective mechanisms constrain the development of expertise specifically required for landscape-scale nature recovery. Without support, nature recovery efforts risk becoming a chimaera: aspirational on paper, but hollow in practice.

### Navigating learning pathways: Between standardisation and flexibility

A second tension concerns the structure of learning and advice within an emerging agri-environmental knowledge system. Contributors described a knowledge landscape marked by patchiness and inconsistency. In the absence of coherent support structures, practitioners have effectively self-curated learning journeys—drawing on peer networks, ad hoc workshops, private consultants, and trial-and-error experiences. Such ‘patchwork learning’ has strengths. It fosters adaptability and innovation grounded in situated knowledge, reflecting the experiential character of knowledge production in complex socio-ecological systems (Collins and Evans 2007; Šūmane et al. 2018). However, the findings here suggest that reliance on informal learning pathways also generates uneven access to expertise, variable quality of advice, and limited opportunities for cumulative learning across networks, as echoed by Thomas et al. (2020) and de Bruin and Ensor (2022). The challenge, therefore, is to balance standardisation (which promotes inclusivity, accountability, and consistent baseline competence) with flexibility (which enables responsiveness to the diverse socio-ecological contexts and ecosystem processes underpinning landscape-scale recovery). Rigid frameworks—especially those tied to compliance or payment structures—risk suppressing innovation and reducing learning to procedural box-ticking (Ayoub 2023). The result is a persistent mismatch between the rhetoric of lifelong learning and the realities of fragmented provision (Šūmane et al. 2018; de Bruin and Ensor, 2022).

These conditions highlight the importance of effective knowledge brokerage. Beyond the provision of technical advice, brokers play an important role in curating learning opportunities, connecting actor networks, and facilitating integration of experiential and formal knowledge (Reed et al. 2014; Cvitanovic et al. 2025), linking experiential and formal learning within AKIS frameworks (de Boon et al. 2022b). Herein, the findings reinforce this role, illustrating how individuals and organisations often act as informal intermediaries linking fragmented knowledge sources. Knowledge brokers—whether individuals, organisations, or informal networks—play a crucial role in connecting actors, translating complex information, and fostering environments where experiential and formal learning intersect (Cvitanovic et al. 2025). Yet, such brokerage functions frequently remain under-resourced or institutionally invisible (MacGregor 2024). Strengthening these connective roles, alongside supporting communities of practice and facilitation capacity, is essential for transforming fragmented learning landscapes into more coherent systems capable of building adaptive capacity for nature recovery.

### Governing for innovation: Entrepreneurial action in a risk-averse system

Interviewees emphasised the need for experimentation, blended finance, and entrepreneurial flexibility to address ecological and market uncertainties at the landscape or catchment scale. Yet, many described governance systems that remain heavily compliance-driven, risk-averse, and bureaucratically rigid. Funding streams often require adherence to pre-defined metrics of success, leaving little room for context-sensitive adaptation or iterative experimentation. This paradox—where innovation is celebrated rhetorically but constrained in practice—penalises those willing to experiment or learn by doing, while privileging actors able to defer engagement until systems stabilise. Thus, the findings highlight the growing importance of entrepreneurial competencies within agri-environmental practice, including opportunity recognition, adaptive business modelling, and risk management. Previous studies have shown that bridging organisations and innovation platforms help address such challenges by creating spaces for collaborative experimentation and knowledge exchange (de Bruin and Ensor 2018; Dias et al. 2019; Pliakoura et al. 2020). Herein, the results reinforce these insights, suggesting that intermediary organisations provided safe-to-fail environments: buffering practitioners against the uncertainties of emergent markets, while facilitating the development of entrepreneurial and collaborative skills. Farmer-led innovation platforms and outcome-based payment schemes exemplify how such capacities can be cultivated, integrating novel financing mechanisms to align ecological restoration with viable livelihood strategies (Klerkx and Begemann 2020; Reed et al. 2022). These models echo insights from innovation and transition studies showing that grassroots and networked innovations often evolve through parallel pathways of experimentation and scaling (Hermans et al. 2016). However, achieving transformative impact will require reconfiguring how risk, accountability, and success are defined—moving from control-oriented compliance to an innovation systems logic that enables adaptive experimentation and continuous learning (Dias et al. 2019; Klerkx and Begemann 2020).

### Bringing the threads together: Towards a reconceptualised A-EKIS

Taken together, these tensions illustrate how the five-skill domains identified in this study interact. Systems thinking and knowledge integration are demanded at the interface of policy and practice. Lifelong learning requires striking a balance between flexibility and coherence in advisory systems. Collaborative partnerships, network building, and agri-environmental entrepreneurship hinge on governance models that recognise brokerage, experimentation, and adaptive business strategies. The work also highlights a series of misalignments (outlined above) that reveal a structural gap between the skills required for landscape-scale transformation and the institutional architectures designed to support them. These observations point towards the need to reconsider how agricultural knowledge and innovation systems are conceptualised in the context of nature recovery, providing the foundation for the reconceptualised Agri-Environmental Knowledge and Innovation System (A-EKIS) outlined below.

The framework extends conventional AKIS approaches by explicitly integrating ecological principles, adaptive governance, and multi-actor knowledge co-production, while foregrounding how learning, coordination, and responsibility unfold across connected landscapes (de Boon et al. 2022a and 2022b; Nettle et al. 2022). Importantly, the analysis suggests that A-EKIS is most appropriately conceptualised at landscape scale, where ecological processes, governance arrangements, and knowledge exchange converge. As such, A-EKIS moves beyond farm-level innovation systems to recognise how the relational and spatial dynamics of learning unfold across interconnected landholdings and communities. In turn, it also recognises the strategic interplay between advice and incentives (Läpple and Hennessy 2015), the transformative and instrumental skillsets required for sustainability transitions (Sørensen et al. 2021; Rastorgueva et al. 2023), and the embedding of justice-oriented, regenerative values within nature recovery efforts (Coulson and Milbourne 2022; Kwauk and Casey 2022). Crucially, the A-EKIS acknowledges that knowledge in land-based systems emerges through formal, informal and non-formal learning, and that adviser and intermediary roles increasingly centre on facilitating cross-boundary collaboration and ecological stewardship at scale.

Together, the findings suggest four interlinked principles through which A-EKIS can be understood:

- **Ecological Embeddedness**: Nature recovery is shaped by ecological variability, landscape histories, and socio-ecological interdependencies that move beyond individual landholdings.
- **Collaborative and Adaptive Learning**: Effective landscape delivery depends on peer exchange, participatory approaches, and reflexive governance, supported by local facilitators, knowledge brokers, and non-traditional experts.
- **Individual and Institutional Connectivity**: Learning and action are shaped by the configuration of policy frameworks, funding structures, advisory networks, and rural institutions in shaping who learns what, and how: thus, linking local action to wider governance scales.
- **Scalar Integration:** Knowledge, coordination, and governance processes unfold across multiple, nested scales—from farm, cluster, catchment, and landscape. Effective knowledge systems must bridge these interfaces to enable collective action and systemic agri-environmental change.

From a practical perspective, this framing provides a foundation for operationalising A-EKIS within landscape-scale policy and delivery contexts. For policymakers, it highlights the importance of aligning incentives, advisory structures, and governance mechanisms to support learning and experimentation across landscapes. For programme leads, it emphasises facilitation, knowledge brokerage, and peer networks in enabling actors to navigate complex change. For advisory organisations, the framework underscores a shift from technical provision towards facilitative roles that support knowledge exchange and adaptive management across diverse actors.

## Conclusions and future perspectives: Towards a skills and knowledge infrastructure for nature’s recovery

This study examined the evolving knowledge and skills for landscape-scale nature recovery in England. Interviews revealed both opportunities and structural challenges in developing, exchanging, and operationalising knowledge at scale. Three interlocking tensions stood out: (1) the gap between policy ambition and institutional capacity; (2) the fragmentation of learning pathways, caught between standardisation and flexibility; and (3) limited scope for entrepreneurial action within compliance-oriented governance frameworks. These reflect deeper structural conditions that inhibit the transformative potential of nature recovery initiatives.

Meeting the challenges highlighted requires a dynamic, multi-sectoral knowledge and skills infrastructure (Burton and Metzger 2022; Raymond et al. 2022): one that integrates technical expertise with adaptive, relational, and entrepreneurial capacities. This reconceptualised A-EKIS must be place-based (responsive to local context), iterative (supporting ongoing learning and feedback), and collaborative by design (bridging disciplines, institutions, and scales) (Reilly et al. 2023; Koch et al. 2025). Strengthening the connective infrastructure requires investment in knowledge brokerage, advisory capacity, and peer-to-peer learning mechanisms for effective exchange and innovation (Adams et al. 2016; Sutherland et al. 2023b; MacGregor 2024). Policy ambition must be matched by governance frameworks that balance standardisation with flexibility, ensuring accountability and locally attuned solutions (Ayoub 2023). Ultimately, embracing uncertainty as a catalyst for adaptive learning and collective action is essential for successful landscape-scale transformation (Kremen and Merenlender 2018; Tedesco et al. 2023). In this sense, the transition towards landscape-scale nature recovery is reshaping what counts as knowledge, who is regarded as an expert, and how learning occurs at greater spatial scales across agri-environmental contexts. The five-domain skills framework and overarching reconceptualisation of AKIS provide a way of holding these dynamics together: acknowledging the stock of technical expertise and connective capacities, and offering a pathway towards a resilient, learning-oriented system that bridges ambition and action to realise the transformative potential of landscape-scale nature recovery.

## Supplementary Information

Below is the link to the electronic supplementary material.

Supplementary file1 (PDF 121 KB)

## Data Availability

The original contributions presented in the study are included in the article and associated Supplementary Information. Further inquiries can be directed to the corresponding first author.

## Abbreviations

AKIS: Agricultural Knowledge and Innovation System
A-EKIS: Agri-Environmental Knowledge and Innovation System
AES: Agri-environment schemes
ELMs: Environmental Land Management Schemes
eNGO: Environmental Non-Governmental Organisation
