# Supplementary material for: Cultivating change: Addressing shifts in knowledge and skills required for landscape-scale nature recovery
Source: Ambio. 2026 Apr 26;55(12):2980–95. doi: 10.1007/s13280-026-02404-z (PMC13731541; doi:10.1007/s13280-026-02404-z)
Supplement: Supplementary file 1 [file 13280_2026_2404_MOESM1_ESM.pdf]

Ambio

Supplementary Information

This supplementary information has not been peer-reviewed:

CULTIVATING CHANGE: ADDRESSING SHIFTS IN KNOWLEDGE AND SKILLS REQUIRED FOR LANDSCAPE-SCALE NATURE RECOVERY

Supplementary Information – Methodological Specification (S1)

Framework Development

The analytical framework was developed through a multi-stage, semi-systematic literature review (see Sørensen et al., 2021; Synder, 2019) conducted between October 2023 and January 2024. This process consisted of: (i) initial generation of keywords and search terms/strings; (ii) literature scoping, data item analysis, and (iii) theme identification. The review examined 80 peer-reviewed research- and review-articles, reports, and policy papers (published in English: 2013-2023) on agricultural extension, sustainability transitions, and environmental governance (databases: Google Scholar, Web of Science, Scopus). Search terms combined variants of '*skills*,' '*knowledge,*' '*agri-environment,*' '*landscape*,' '*nature recovery*,' and '*learning systems*,' as evidenced below:

TS=((barrier* OR challenge* OR opportunit*) AND (Knowledge* OR skill* OR competenc*) AND (sustainab* OR agricultu* OR agri-env* OR nature-based* OR restor*) AND (farmer* OR land manager* OR extension* OR advisor*))

The initial literature search generated 8,405 results. Of these, 492 titles were screened, and 404 papers assessed for relevance. After staged exclusions, 65 research articles remained for detailed analysis (30 on skills acquisition and development; 35 on broader agri-environmental and

landscape-scale transition). Additionally, 15 review articles were used to contextualise the research, but were excluded from the thematic dataset. Following this PRISMA-style workflow, literature was screened in three stages: (1) title and abstract review, (2) full-text assessment, and (3) structured thematic extraction. During stage three, each article was coded for explicit references to knowledge, skills, practices, and enabling conditions for environmental or agricultural transition. Codes were iteratively grouped into higher-order categories based on conceptual similarity and recurrence. From this, five interrelated skill domains were derived — systems thinking, lifelong and life-wide learning, collaborative partnerships and network building, agri-environmental entrepreneurship, and applied expertise and knowledge integration. These domains reflect the frequently identified and theoretically supported competencies associated with ecological transition. This framework was then refined and validated during the exploratory interview phase through iterative coding and cross-comparison, ensuring alignment with lived stakeholder experience.

Ethical & Reflexive Considerations

Ethical approval was obtained from the University of Gloucestershire's CCRI Ethics Panel (Ref: CCRI_002; CCRI_002_ext1). All participants gave written and verbal consent, and data were anonymised to ensure confidentiality. A reflexive log was maintained throughout data collection and analysis to track interpretive decisions and evolving assumptions. Nevertheless, findings are necessarily partial and time-bound, reflecting policy and sectoral shifts during the 2024–25 transition period. The sample is geographically limited (England and Wales), and despite inclusion of diverse roles, certain groups - such as tenants, minoritised communities, and early-career practitioners - remain underrepresented. Furthermore, greater attention could be attributed to landscape history and characteristics. While the skills framework supports comparative analysis, it inevitably oversimplifies the fluidity of practice. These limitations are acknowledged as opportunities for further research. Nonetheless, the diversity of institutional contexts provides a strong foundation for identifying emerging patterns, tensions, and leverage points for policy and practice.

Relevant Citations:

Snyder, H. (2019). Literature review as a research methodology: An overview and guidelines. *Journal of Business Research*. 104: 333-339. https://doi.org/10.1016/j.jbusres.2019.07.039

Sørensen, L.B., L.B. Germundsson, S.R. Hansen, C. Rojas, and N.H. Kristensen. 2021. What Skills Do Agricultural Professionals Need in the Transition towards a Sustainable Agriculture? A Qualitative Literature Review. *Sustainability.* 13 (24): 13556. https://doi.org/10.3390/su132413556

Appendix S2 – Example Thematic Guide (Knowledge User):

Introduction & Background

1. What is your background & current role? How would you describe your occupation?

2. Are you the owner (or involved in the management) of land for agricultural, conservation, or nature restoration purposes?

3. (If relevant) Can you tell me a little about the farm/landholding/project (i.e., Type, historic management, ownership/tenure, size, location, etc)?

4. (If relevant) Across the farm/landholding(s) you're involved with and/or interested in, are any undertaking formal land management agreements/voluntary schemes related to agri-environmental practice?

Individual Perceptions of Nature Recovery

UK land management is evolving, driven, in part, by changes in climate, agriculture, and public policy. More specifically, there are wide-ranging changes in relation to subsidy schemes (i.e., post-Brexit phasing out of basic payments and introduction of environmental land management schemes) alongside novel regulations, trade agreements, and institutional support, etc.

5. What do you make of these changes, and how does this impact you, if at all?

6. Where do you believe responsibility for this transition lies, and at what scale?

As the agricultural sector transitions towards prioritising environmental objectives alongside (or in place of) diversified agri-food systems, it would seem to many that global notions of the 'good farmer' are increasingly centred on nature-inclusive land and resource management, or nature recovery.

7. How do you understand and approach ‘nature recovery’ in the context of your work?

8. What are the primary driver(s) of change influencing nature recovery efforts?

9. (If relevant) What are the main barriers or challenges hindering the adoption of nature recovery practices at scale?

10. Do you feel that existing policies and support mechanisms adequately address practitioners’ needs regarding nature recovery?

11. Are there any untapped opportunities or potential incentives that could encourage greater engagement in nature recovery?

<u>Skills Acquisition & Development for Nature Recovery</u>

12. What skills do you believe are required for nature recovery?

13. Do you feel adequately equipped with the necessary skills and knowledge to engage in nature recovery initiatives?

14. Have you received any formal training or education related to nature recovery? If so, what did this entail?

15. What do you make of the general availability, level, and quality of current learning/training opportunities in the land-based sector, specifically those centred around agri-environmental issues such as nature recovery?

16. Where would you look to find suitable training opportunities? What have you seen offered?

17. What types of training or educational/capacity-building programs would be most beneficial for enhancing skills related to nature recovery? How should these be delivered?

18. How can institutions better support individuals and organisations in developing the necessary skills for nature recovery? How could this benefit you, if at all?

<u>Sign Off & Participant/Case Study Mapping</u>

The latter stage of this research involves a series of tailored surveys and a selection of case studies of landscape-scale, nature-based recovery (encompassing a range of farming and environmental contexts, business/financing models, skills requirements, learning pathways, and broader motivational backgrounds) to provide an inquiry into the skills required (and already acquired) by stakeholders undertaking large-scale changes in land use and management.

19. Do you know of any suitable initiatives/examples in England that might be suitable? Are there any individuals, farming/lifestyle landholder networks, DEFRA pilot projects, or organisations you know of that could be helpful for me to get in touch with or that you could refer me to?

20. Is there anything else you would like to share regarding skills needs, innovation requirements, or other aspects of nature recovery that have not been covered?

____

Appendix S3 – Example Thematic Guide (Knowledge User):

Introduction & Background

1. What is your background & current role? How would you describe your occupation?

2. (If relevant) What is the size & scope of your organization? Is it involved in the ownership or management of land for agricultural, conservation, or nature restoration purposes?

3. (If relevant) Across landholdings associated with the organisation, are any involved within formal agreements/voluntary schemes related to nature recovery, conservation or agri-environmental practice? What changes has that entailed?

Individual Perceptions of Nature Recovery

UK land management is evolving, driven, in part, by changes in climate, agriculture, and public policy. More specifically, there are wide-ranging changes in relation to subsidy schemes (i.e., post-Brexit phasing out of basic payments and introduction of environmental land management schemes) alongside novel regulations, trade agreements, and institutional support.

4. What do you think of these changes, and how do you believe this impacts farmers/land managers?

5. Where do you believe responsibility for this transition lies, and at what scale?

As the agricultural sector transitions towards prioritising environmental objectives alongside (or in place of) diversified agri-food systems, global notions of the 'good farmer' are increasingly centred on nature-inclusive land and resource management, or nature recovery.

6. How do you understand/define ‘nature recovery’ in the context of you/your organisations’ work?

7. What are the key driver(s) of change influencing nature recovery efforts?

8. (If relevant) What are the main barriers or challenges hindering the adoption of nature recovery practices at landscape scale?

9. Do you feel that existing policies and support mechanisms adequately address practitioners’ needs regarding nature recovery?

10. Are there any untapped opportunities and/or potential incentives that could encourage greater engagement in nature recovery?

<u>Skills Acquisition & Development</u>

11. Have you received any formal training or education related to nature recovery? If so, what did this entail?

12. What skills do you believe are required for nature recovery?

13. From your experience, are knowledge providers sufficiently skilled to consider agri-environmental issues such as nature-based recovery?

14. Do you feel that farmers/land managers are generally well equipped with the necessary skills and knowledge to engage and participate in nature recovery?

15. What do you make of the general availability, level, and quality of current learning/training opportunities in the land-based sector: specifically, those centred around issues such as nature recovery?

16. What types of training or educational/capacity-building programs do would be most beneficial for enhancing skills related to nature recovery?

17. If interested, where do farmers/land managers find suitable training opportunities? What have you seen offered?

18. How can institutions and organisations best support individuals and organisations in developing the necessary skills for nature recovery?

<u>Sign Off & Participant/Case Study Mapping</u>

The latter stage of this research involves the selection of case studies of landscape-scale, nature recovery initiatives (encompassing a range of farming and environmental contexts, business/financing models, skills requirements, learning pathways, and broader motivational backgrounds) to provide an inquiry into the skills required, and already acquired, by stakeholders undertaking large-scale changes in land use and management.

19. Do you know of any suitable initiatives/examples in England that might be suitable? Also, are there any individuals, farming/lifestyle landholder networks, DEFRA pilot projects, or

organisations you know of that could be helpful for me to get in touch with or that you could refer me to?

20. Is there anything else you would like to share regarding skills needs, innovation requirements, or other aspects of nature recovery that have not been covered?
